# Socio-Economic Drivers of Renewable Energy: Empirical Evidence from BRICS

**DOI:** 10.3390/ijerph19084614

**Published:** 2022-04-11

**Authors:** Usman Mehmood, Ephraim Bonah Agyekum, Salman Tariq, Zia Ul Haq, Solomon Eghosa Uhunamure, Joshua Nosa Edokpayi, Ayesha Azhar

**Affiliations:** 1 Remote Sensing, GIS and Climatic Research Lab, National Center of GIS and Space Applications, Centre for Remote Sensing, University of the Punjab, Lahore 54590, Pakistan; zia.spsc@yahoo.com (Z.U.H.); ayesha.rsgcrl@pu.edu.pk (A.A.); 2 Department of Political Science, University of Management and Technology, Lahore 54770, Pakistan; 3 Department of Nuclear and Renewable Energy, Ural Federal University, Mira 19, 620002 Ekaterinburg, Russia; agyekumephraim@yahoo.com; 4 Remote Sensing, GIS and Climatic Research Lab, National Center of GIS and Space Applications, Department of Space Science, University of the Punjab, Lahore 54590, Pakistan; salmantariq_pu@yahoo.com; 5 Faculty of Applied Sciences, Cape Peninsula University of Technology, P.O. Box 652, Cape Town 8000, South Africa; uhunamures@cput.ac.za; 6 Faculty of Science, Engineering and Agriculture, University of Venda, Private Bag X5050, Thohoyandou 0950, South Africa; joshua.edokpayi@univen.ac.za

**Keywords:** BRICS countries, CO_2_ emissions, clean environment, sustainable development, renewable energy

## Abstract

There is a need to implement efficient strategies to mitigate the challenges of climate change and income inequalities in developing countries. Several studies have been conducted to address the relationship among different econometric and environmental indicators of renewable energy (RE) but overlooked the relationship between RE and income inequalities. This study investigates the influence of the distribution of income on the RE in Brazil, Russia, China, and South Africa (BRICS) between 1988 and 2017. The econometric (economic growth and trade), environmental, and institutional parameters are also integrated into the model. The outcomes reveal that reduced inequality in income distribution increases the consumption of RE. In contrast, CO_2_ emissions have a positive correlation with RE. The governments should implement environmentally friendly policies and increase the consumption of renewable energy in the future with regards to reducing environmental pollution. Furthermore, findings from the study indicate a positive effect on the reduction of corruption in renewable energy. This shows that institutional quality can affect the uptake of renewable energy. The study further identified that growth in a country’s economy decreases RE consumption, suggesting that these countries prefer fossil fuels to gain economic growth. The Granger causality results show that a bidirectional causality exists between income inequality and RE consumption. Bidirectional causality is observed between income distribution and CO_2_ emissions. The results from this study are important for policymakers to achieve sustainable development because fair income distribution and environmental quality are considered as two key factors for sustainable development. Strong institutions and control on corruption can bring sound social and economic gains. Therefore, fair distribution of income and strong institutional policies can increase RE consumption to achieve a clean environment.

## 1. Introduction

The social, economic, and ecological life of future generations requires some efficient decisions today. Therefore, the world is striving to achieve sustainable development. In 2015, 193 countries resolved to achieve sustainable development goals [1]. This plan aims reducing poverty, inequality, and improve environmental quality. It is often debated in international forums that environmental pollution and income inequality are making obstacles in the way of achieving sustainable development goals [2]. Therefore, the United Nations Organization (UNO) pointed out the importance of efficient strategies to mitigate the problems of climate change and inequalities in all nations [3,4].

Sachs [5] argued that countries have made progress in terms of the growth of their economies, but most have failed to address the problems of establishing welfare societies and environmental pollution. To establish harmonious societies, the most important obstacle is income inequality. Since the last decades, the world has been facing the rapid deterioration of income distribution [6]. This hasty worsening of income allocation has attracted scholarly attention in examining its dynamics in developed and developing countries [7]. Along with the developed world, income distribution is rapidly worsening in developing countries.

Environmental degradation is also an obstacle to achieving sustainable development goals. To achieve more economic growth, the developed and developing countries are consuming natural resources without considering the environmental effects. Rapid population growth and the growing role of globalization is depleting fossil fuels [8]. According to recent reports by BP statistical assessment, 75% of the world’s leading energy consumption is generated from crude oil, coal, and natural gas. The use of fossil fuels lowers the efficiency of energy and creates environmental crises by emitting greenhouse gasses such as carbon dioxide (CO_2_). Despite the collective efforts of the world to reduce the concentration of CO_2_ emissions, it continues to increase rapidly. Therefore, to achieve sustainable development, it is essential to reduce the concentration of CO_2_ emissions. In this scenario, renewable energy has become an important alternative source to compensate for the energy requirements to achieve sustainable growth [9]. Renewable energy is a viable source of energy due to its lower CO_2_ emissions as compared to non-renewable energy resources. Shahbaz et al. [10] argued that the emissions of CO_2_ are linked to the use of non-renewable energy resources, and it is imperative to consume renewable energy resources to reduce environmental pollution.

Due to irregular changes in oil prices and energy protection issues, the world is shifting its energy demands towards renewable energy resources [11]. The consumption of renewable energy has increased from 2007 to 2017 by 16.4% but this share is still low as it only accounts for 4% of the total energy consumption. In this context, it is very important to find out those aspects that can raise the share of clean energy in terms of environmental quality.

### Research Gap

In this manner, the increasing concern over environmental pollution and the rising problem of inequality in the distribution of income has created a new aspect to investigate linkages between climatic pollution with socioeconomic issues. Therefore, it is imperative to investigate whether income inequality has an impact on environmental quality or not [12]. Many researchers now examine the impact of income inequality on ecological indicators, but there has not been harmony on the experimental level. Some argued that climatic issues are initiated from income and authoritative inequalities, but some intellectuals are of the view that income inequality may increase or may not affect environmental pollution. Although there is enough literature that provided the link between inequality in income and renewable energy, even though the shock of inequality in the distribution of income on clean energy is ignored. The recent studies which find the key drivers of renewable energy are Torras and Boyce [13] and Uzar and Eyuboglu [14]. Among the available studies, which probe the environmental, economic, and political determents of clean energy, the social aspect of economic unfairness has not been fully exploited. Clean energy helps in improving air quality, but it is affected by income inequalities. For instance, when fair income prevails in any society it may boost the concerns of the society for a cleaner environment. Therefore, the demand for cleaner air may affect the demand for renewable energy. In this regard, it is important to probe the potential association between renewable energy and income inequality regarding environmental pollution.

This research strives to find the effects of economic unfairness on renewable energy sources in BRICS nations using the available data of 1988–2017. The study also includes economic (trade openness, GDP), institutional (corruption) and environmental indicators (emission of CO_2_). Thus, a comprehensive study is conducted to find the possible drivers of renewable energy in the BRICS nations.

This research intends to also add to the existing body of literature in several new ways. Even though there exist sufficient studies which found the causal factors of renewable energy, there is a dearth of studies conducted based on country-specific analysis to find the income inequality-renewable energy nexus. For example, Apergis [15] investigated the effect of clean energy on economic inequality, McGee and Greiner [16] investigated the consequences of economic inequality and renewable energy on the emission of CO_2_. Therefore, no country-specific study has been conducted to probe the effect of income inequality on renewable energy. The empirical findings of this study can be very significant for policymakers to form energy and environmentally friendly policies. Policymakers can have the opportunities to design harmonious societies to improve air quality if a negative relationship is found by this study.

This study is structured as follows: the literature review is indexed as Section 2, the methodology as Section 3, the discussion as Section 4 and the conclusion as Section 5.

## 2. Literature Review

According to Laurent [17], there are three pillars of sustainable development, namely; economic, ecological and social. Moreover, economic-social and economic-ecological connections have been analyzed but the social-ecological connection is still vague. Therefore, there are very few studies on this subject that lack theoretical background from a potential link between renewable energy, economic and inequality that can be traced from the past studies on income inequality and climate.

Berthe and Elie [18] performed a comprehensive study to probe the potential link between economic inequality and clean energy by providing sound background literature between both variables. However, Uzar [19] used the practical method of Berthe and Elie [18] to examine the possible connection among the estimated parameters.

According to Uzar [19], individual economic preferences can affect environmental quality through consumerism. Boyce [20] stated that societies with greater income inequalities compete for social status. In doing so, some consume to maintain their status, and some consume to increase their status. In such circumstances, when production has increased, the maintenance of the machinery affects the costs of the goods. As a result, the prices of goods increase which make it difficult to maintain production. Comparatively, renewable energy is more costly (i.e., initial cost) than fossil fuels. Particularly as there is a lack of awareness in the society especially for the people in the low-income bracket. Therefore, preference is given to traditional means i.e., fossil fuels without considering the cost and affordability of renewable energy to be used.

Consequently, a society with more income inequality cannot predict the long-term negative effect of fossil fuels. The poor can believe that economic escalation can lift their status in society. In this regard, economic profit may overcome environmental benefits. According to Laurent [17] and Berthe and Elie [18], Europeans and Americans have shown more concerns about employment and economic growth than environmental problems. Thus, individualism, consumerism and short-termism may shape the utilization of renewable energy in discordant societies.

According to Mehmood [21], Pakistan, India, Bangladesh, and Sri Lanka need to revise their international trade policies to reduce carbon dioxide emissions. Tariq et al. [22] explored environmental Philips’s curve for South Asian countries. It was found out that there was economic sustainability by increasing the share of renewable energy sources. Moreover, there are research studies that focused on the importance of institutions in determining climatic policies [23,24]. Institutional factor such as corruption is perhaps the important aspect in presenting the link between economic inequality and climatic pollution. Income inequality can be a factor of a weak government. According to a study by Wolde-Rufael and Idowu [12], corruption can weaken strict ecological regulations. Similarly, Fredriksson and Svensson [25] argued that corruption can hamper competent investments in clean energy projects.

Since the last few years, the problems of income inequality and environmental pollution have attracted worldwide attention. Most studies have focused on the impacts of economic inequality on ecological factors like emission of CO_2_, water pollution, environmental footprints, and air pollution. A review of these studies indicated heterogeneity based on the obtained empirical results.

Torras and Boyce [13], researched on 58 countries and found that income inequality affects environmental pollution negatively. Golley and Meng [26] provided evidence that reducing the inequality in China significantly reduced the rate of household emissions. In the United States of America, Baek and Gweisah [27] investigated the nexus between unfairness in income and CO_2_ emissions. The result found that fair income distribution reduces the emissions of CO_2_ in the long and short-run dynamics. A study by Jorgenson [28] indicated that inequality in income increases the intensity of CO_2_ released for OECD and non-OECD countries. Liu et al. [2] analyzed obtained data of 1997–2015 for the USA and establish that income distribution positively affects CO_2_ emissions in the short-run, but negatively in the long run. Uzar and Eyuboglu [14] analyzed the effect of income inequality on the emissions of CO_2_ in Turkey. The result shows that improvements in income inequality lower the emission of CO_2_.

Some studies, however, could not find a significant association between income inequality and environmental pollution. Scruggs [29] conducted a study for a group of economies and revealed that income distribution has no significant impact on environmental quality. Ravallion et al. [30] studied the effect of income disparities on CO_2_ emissions for different countries from 1975 to 1992 and showed that economic inequality condensed CO_2_ emissions. In another study by Wolde-Rufael and Idowu [12], the relationship between income inequality and emission of CO_2_ for India and China was probed over the periods of 1971–2010 and 1974–2010, respectively. Statistically, no significant connection between the two variables was found. Unlike Liu et al. [31], Wolde-Rufael and Idowu [12] observed the significant role of inequality in the distribution of income in improving environmental quality.

The role of renewable energy is debated widely for its environmental friendly effects [32]. The probability of renewable energy to improve air quality has led the research community to find its possible determinants for different countries. From the literature, available studies have probed the association between GDP, emission of CO_2_, employment, trade openness, and energy prices. Additionally, some research studies have been done to investigate the political factors of renewable energy [23,24,33]. Sadorsky and Perry [34] computed the annual data obtained from 1980 to 2005 for G7 countries and found that emission of CO_2_ and income (per capita) are the key drivers for renewable energy. From examining the relationship between renewable energy and real GDP in twenty OECD nations, Apergis and Payne [35] established the two-way causality between the variables. In a study by Rafiq et al. [36], the connection between the emission of CO_2_, GDP, and renewable energy for China and India was evaluated. The study found a bidirectional causality among the three variables in India but bidirectional causality between renewable energy and emission of CO_2_ resources in China. Apergis and Payne [37] investigated the connection between economic and environmental features for seven African economies. The result indicated a positive relationship between the variables (emission of CO_2,_ GDP, and renewable energy). Ben Jebli and Ben Youssef [38] probed the relationship between renewable and non-renewable energy, CO_2_ emissions, GDP growth, and trade openness for the years 1980–2009. The study found the unidirectional causality among the dependent variables and renewable energy.

Few studies have paid attention to the political factors of renewable energy. For example, Cadoret and Padovano [23] argued that corruption control positively affects renewable energy while lobbying decreased renewable energy production in European countries. Sequeira and Santos [24] observed that democracy increases renewable energy resources. Hence, there is extensive literature available, which find the political, economic, environmental determinants of renewable energy, but studies that probe the economic unfairness of renewable energy consumption linkages are quite a few in number. Among those studies, Apergis [15], attempted to correlate the association between renewable energy and income inequality for 32 OECD nations during the years 1998 to 2013. The impact on income inequality was statistically positive and robust across the different types of renewable energy sources. Recently, Uzar [19] performed a panel study for 43 countries to study the impact of income inequality on renewable energy and found that improved distribution of income will enhance the consumption of renewable energy. However, the panel study can have some limitations to provide policy instruments for specific countries [19]. This research, therefore, is a novel attempt that investigates the linkages between economic inequality and renewable energy for BRICS countries.

## 3. Data and Methodology

Equation (1) is the estimated equation formed after the time series were transformed to logarithmic form.

(1) $$RE_{it\;}=\beta_{0\;}+\beta_{1\;}GINI_{it\;}+\beta_{2\;}G_{it\;}+\beta_{3\;}COR_{it\;}+\beta_{4\;}TR_{it\;}+\beta_{5\;}CO_{2it\;}+\epsilon_{it\;\;\;\;}$$

In Equation (3) the constant term is $\beta_{0\;}$ and $\beta_{1\;}$, $\beta_{2\;}$, $\beta_{3\;}$, $\beta_{4\;}$, $\beta_{5\;}$ are coefficient of variables $\epsilon_{it\;}$ is error correction term (ECT). RE represents renewable energy, which is in Terawatt hours. Renewable energy consists of solar, wind power, geothermal and biomass generation. Renewable energy and CO_2_ emissions data were obtained from the BP statistical assessment of world energy (2019). GINI is the main independent variable, which represents income inequality. The data for income inequality was obtained from the global standardized income-inequality datasets of Solt [39]. The GINI index lies from 1 to 100. The higher value shows a worsened condition of the income distribution. G is GDP (constant 2010$) represent economic growth and TR is the trade openness. Both parameters are downloaded from the global data indicators. Lastly, the data for corruption (COR) was obtained from the ICRG of PRS group. ICRG ranks the countries on a scale of 0 to 6. Maximum value means a lower level of corruption and minimum value means a higher level of corruption. The corruption variable was included to catch the institutional factor in the model.

Although Uzar [19] has analyzed the associations of inequality in the distribution of income and renewable energy from panel data of 43 nations worldwide. However, no study has been conducted for BRICS nations, which gap this study seeks to fill.

To ensure constant variation, all variables are transformed into logarithmic form and the panel Pooled Mean Group (PMG) method developed by Pesaran et al. [40] was applied. This technique has been accompanied by an error-correction model and is an efficient approach to know long-run and short-run coefficients [19]. Moreover, PMG technique is superior to other approaches to provide long-run outcomes. Furthermore, the parameters do not have to be stationary at 2nd difference. Secondly, PMG technique removes the problem of endogeneity by incorporating the length of lags of both dependent and independent parameters to give a robust outcome [19]. Thus, PMG-model can be as follows:

(2) $$lnRE_{it}=\;\overset{p}{\underset{n=1}{\sum }}\partial_{n}lnRE_{i\left(t-n\right)\;\;}+\;\;\;\;\;\;\overset{q1}{\underset{n=0}{\sum }}\delta_{o}lnX_{i\left(t-n\right)\;\;}+\gamma_{i}+\varepsilon_{it}\;\;\;\;\;\;\;\;\;$$

In below Equation (3), X denotes the independent parameter:

(3) $$∆{lnRE}_{it}=\alpha_{i}\left(lnRE_{i\left(t-1\right)}-\delta_{i}X_{i}\right)+\overset{p-1}{\underset{n=1}{\sum }}\partial_{n}∆lnRE_{i\left(t-n\right)\;\;}+\;\;\;\;\;\;\overset{q1-1}{\underset{n=0}{\sum }}\delta_{o}∆lnX_{i\left(t-n\right)\;\;}+\gamma_{i}+\varepsilon_{it}\;\;\;\;\;\;$$

In Equation (3), X denotes the independent vector of parameters of $lnGINI_{it}$, $lnCOR_{it}$, $lnG_{it}$, $lnTR_{it}$, and $lnCO_{2it}$. $\delta_{i}$ shows the value of the estimated long-run coefficient of health expenditures. $\alpha_{i}$ characterizes the error correction term. $\varepsilon_{it}$ represent the error value in the independent distribution of the data. $\gamma_{i}$ represents the group effect. The rest of the terms shows coefficients of short-run estimates. Before the PMG technique, it is mandatory to apply the unit root test. After the unit root test, long-run and short-run analyses were carried out.

## 4. Results and Discussion

Firstly, establishing the integration order among the parameters as indicated in Table 1 and Table 2 provide the results of the 1st generation and 2nd generation unit root tests. These unit root tests are reliable and provide robust results [41]. It is noted that almost all the parameters are integrated at 1st difference in both sets of unit root tests. This means that almost all the variables are moving together in the long run. After checking the integration order, the Pedroni co-integration test was performed to verify the long-run association. This test provides robust findings by considering the cross-section dependence among the panel data.

Table 3 presents the outcomes of the Pedroni co-integration test, which is indicating that the variables are correlating collectively in the long run at 1% level of significance. To uncover the short and long-run associations, a PMG estimator was performed as presented in Table 4. It can be noted that the GINI coefficient is negatively correlated with renewable energy in the long run. This means fair income distribution increases renewable energy use. The equal distribution of income provides equal economic opportunities to the people, as they start to think positively towards their climate. Environmental care lowers the usage of non-renewable energy sources as people tend to use cleaner energies. These results are quite similar to a study conducted by Uzar [19]. There can be justifications about the positive impact of fair distribution of income on renewable energy. For instance, in any society with equal income distribution, individuals can demand a clean environment. This is because fair income will reduce individuality and produce mutual awareness about the use of renewable energy for a clean environment. In such a context, governments can invest in renewable energy projects by offering economic incentives.

Moreover, a fair distribution of income will reduce the lobbying of elite groups in society, who can affect the renewable energy consumption policies by flexible regulations. GDP is negatively linked with renewable energy, but it is insignificant, implying that these countries are using non-renewable energies to gain more economic growth at cost of the environment. The role of trade openness is increasing renewable energy consumption, which means that these countries should promote trade activities with other countries. Trade is positively associated with renewable energy in BRICS countries. The error correction term is denoted by ECT as presented in Table 4.

Similarly, CO_2_ emissions are positively affecting renewable energy in BRICS nations, which means that increase in CO_2_ emissions will increase the ratio of renewable energy in these nations. There can be an argument to support this finding. For example, fossil fuels contribute towards more pollution and thus there is the need for governments to implement strict environmental regulations to lower its consumption and promote non-renewable energy. This will make the people utilize clean energy at domestic and commercial levels by exploring the available renewable energy sources to replace fossil fuels.

To capture the impacts of institutional factors, this study includes the corruption control variable, which was positively associated with renewable energy consumption. Corruption control was positively affecting renewable energy in the long run. This means that the reduction in corruption will increase the consumption of renewable energy. These results are in line with the results obtained by Uzar [19], which investigated the role of different institutional factors in ringing renewable energy consumption. The corrupt official can prevent environmental protection projects for their gains. Therefore, strong institutions with lower corruption will surely increase strict environmental policies. The results obtained from Dumitrescu and Hurlin [42] causality test are given in Table 5. The result indicated a bidirectional causality exists among income inequality, COR, GDP, trade, and renewable energy. Fairer income distribution will increase renewable energy consumption and renewable energy consumption will assist in reducing income inequality in BRICS nations. Renewable energy is also causing economic growth [43]. These findings are in line with the study of Usman and Hammar [44] which found a bidirectional causal linkage between RE and G. In this context, BRICS nations can improve their economic growth sustainably if they reduce income inequality. The GDP is lowering renewable energy consumption, which means that these countries are still relying on non-renewable energy sources for rapid economic growth. These findings are important for policymakers to consider all estimated variables to enhance the ratio of renewable energy in BRICS nations.

## 5. Conclusions

In this study, the potential effect of income distribution on renewable energy in BRICS countries was analyzed from 1988 to 2017. The investigation shows that there are some major shortcomings in literature; (i) correlation was taken as causation; (ii) the estimation impact of RE on CO_2_ or vice versa; (iii) renewable energy is linked with income inequality. For this, the study integrated econometric, institutional, and environmental parameters such as trading, economic growth, and corruption in the model. The results reveal positive impacts of emission of CO_2_ on renewable energy and reduction in corruption due to strong institutional quality. Moreover, lessening inequalities in the income distribution also increase in renewable energy. BRICS countries prefer the use of fossil fuels to meet the pace of economic growth as it decreases the consumption of renewable energy. The BRICS countries should promote trade and the use of renewable energy resources. The findings of this study validate that, by lowering income inequality, BRICS nations can increase their share of renewable energy consumption. This means that income equality raises environmental awareness and people start to demand strict environmental regulations. Strict environmental regulations make the government produce renewable energy rather than consuming energy from non-renewable energy sources. The GDP is lowering renewable energy consumption, which means that these countries are still relying on non-renewable energy sources for rapid economic growth. The outcome from this study shows that BRICS countries should explore the available renewable energy sources to replace the dependence on fossil fuels. The Granger causality test indicated that a bidirectional causality exists among CO_2_ emissions, income inequality, and consumption of renewable energy. This analysis will help in the understanding of the simultaneous impact of variables on each other.

This study also presents some very important policy implications for BRICS countries which will help in the achievement of Sustainable Development Goals. Firstly, economic factors are determining the consumption of renewable energy to improve air quality. Apart from economic factors, the institutional, environmental, and socioeconomic factors are also contributors towards renewable energy production. To achieve sustainable development, this study reveals that fair distribution of income and quality of the environment are considered as two most important drivers. These two objectives can be achieved in chorus. Strong institutional quality and corruption control can bring sound socio-economic benefits to the BRICS countries. Therefore, fair income distribution and strong institutions can increase renewable energy consumption to achieve a clean environment. Considering the important policy instruments presented by this study, future studies should be conducted for South Asian and the G7 countries.

## Figures and Tables

**Table 1:** First-generation unit root test.

Variable	Levin-Lin-Chu Unit Root Test		Im, Pesaran, Shin	
	At Level	1st Difference	At Level	1st Difference
${lnRE}_{it}$	1.22	−2.30 ***	2.69	−1.87 **
${lnGINI}_{it}$	−1.21	−2.81 ***	1.05	−1.91 **
${lnCOR}_{it}$	−1.23	−5.52 ***	−0.71	−4.98 ***
${lnCO}_{2it}$	0.02	−3.71 ***	−0.39	−4.45 ***
${lnG}_{it}$	−4.89 ***	−3.73 ***	−2.52 **	−2.72 ***
${lnTR}_{it}$	−2.48 ***	−5.17 ***	−0.34	−4.47 ***

Note: ** and *** represent significant levels of 5% and 1%, respectively

**Table 2:** Second Generation unit root test.

Variable	CIPS Unit Root Test		CADF Unit Root Test	
	At Level	First Difference	At Level	First Difference
${lnRE}_{it}$	−1.90	−2.40 **	−1.72	−2.70 **
${lnGINI}_{it}$	−0.88	−1.54 *	−1.42	−2.84 **
${lnCOR}_{it}$	−0.76	−3.12 ***	−1.32	−2.25 **
${lnCO}_{2it}$	−1.01	−3.12 ***	−1.89	−3.32 ***
${lnG}_{it}$	−1.37	−2.11 ***	−1.68	−2.78 **
${lnTR}_{it}$	−1.51	−2.35 ***	−3.13 ***	−3.46 ***

Note: *, ** and *** represent significant levels of 10%, 5% and 1%, respectively.

**Table 3:** Pedroni co-integration test.

	Statistics	Prob.	Statistics	Prob.
Panel v-Statistic	3.028334	0.0012	1.657198	0.0487
Panel rho-Statistic	2.632656	0.9958	2.460050	0.9931
Panel PP-Statistic	−5.580482	0.0000	−7.392751	0.0000
Panel ADF-Statistic	−3.202189	0.0007	−2.213418	0.0134
Group rho-Statistic	3.354774	0.9996		
Group PP-Statistic	−9.167237	0.0000		
Group ADF-Statistic	−2.608589	0.0045		

**Table 4:** PMG estimation.

Variables	Coefficients	Std. Error	T-Statistics	*p*-Value
$lnGINI_{it}$	−3.31 ***	1.25	−2.63	0.01
$lnCOR_{it}$	3.35 ***	0.87	3.79	0.00
$lnCO_{2it}$	0.12 **	0.40	0.31	0.07
$lnG_{it}$	−0.27	0.31	−0.86	0.10
$lnTR_{it}$	0.74 **	0.33	2.23	0.03
Short-run				
ECT^−1^	−0.11 **	0.06	−1.71	0.09
$lnGINI_{it}$	24.14	23.74	1.01	0.31
$lnCOR_{it}$	−0.08	0.79	−0.11	0.91
$lnCO_{2it}$	0.79	1.38	0.57	0.57
$lnG_{it}$	−3.03	2.47	−1.22	0.22
$lnTR_{it}$	1.16	0.82	1.41	0.16

Note: ** and *** represent significant levels of 5% and 1%, respectively.

**Table 5:** Causality results.

Null-Hypothesis	WStat.	ZbarStat.	Prob. Value
GINI→RE	4.16 ***	3.33	0.00
RE→GINI	5.23 ***	4.53	0.00
G→RE	6.45 ***	5.90	0.00
RE→G	2.67 **	1.66	0.09
COR→RE	1.51	0.35	0.79
RE→COR	1.73	0.59	0.57
${\mathrm{CO}}_{2}\to \mathrm{RE}$	4.51 ***	3.72	0.00
$\mathrm{RE}\to {\mathrm{CO}}_{2}$	3.53 ***	2.62	0.00
TR→RE	4.62 ***	3.84	0.00
RE→TR	2.82 **	1.82	0.03

Note: ** and *** represent significant levels of 5% and 1%, respectively.

## Data Availability

All data used are available in the text.
